# The relationship, structure and profiles of schizophrenia measurements: a post-hoc analysis of the baseline measures from a randomized clinical trial

**DOI:** 10.1186/1471-244X-11-203

**Published:** 2011-12-28

**Authors:** Lei Chen, Glenn Phillips, Joseph Johnston, Bruce J Kinon, Haya Ascher-Svanum, Sara Kollack-Walker, Paul Succop, Dieter Naber

**Affiliations:** 1 Lilly & Company, Lilly Corporate Center, Indianapolis, IN, 46285 USA; 2 Sunovion Pharmaceuticals, Inc., Marlborough, MA, 01752 USA; 3 Lilly USA, LLC, Indianapolis, IN, 46285 USA; 4 University of Cincinnati, Cincinnati, OH, 45221 USA; 5 University Medical Center Hamburg-Eppendorf, Hamburg, Germany

## Abstract

**Background:**

To fully assess the various dimensions affected by schizophrenia, clinical trials often include multiple scales measuring various symptom profiles, cognition, quality of life, subjective well-being, and functional impairment. In this exploratory study, we characterized the relationships among six clinical, functional, cognitive, and quality-of-life measures, identifying a parsimonious set of measurements.

**Methods:**

We used baseline data from a randomized, multicenter study of patients diagnosed with schizophrenia, schizoaffective disorder, or schizophreniform disorder who were experiencing an acute symptom exacerbation (n = 628) to examine the relationship among several outcome measures. These measures included the Positive and Negative Syndrome Scale (PANSS), Montgomery-Asberg Depression Rating Scale (MADRS), Brief Assessment of Cognition in Schizophrenia Symbol Coding Test, Subjective Well-being Under Neuroleptics Scale Short Form (SWN-K), Schizophrenia Objective Functioning Instrument (SOFI), and Quality of Life Scale (QLS). Three analytic approaches were used: 1) path analysis; 2) factor analysis; and 3) categorical latent variable analysis. In the optimal path model, the SWN-K was selected as the final outcome, while the SOFI mediated the effect of the exogenous variables (PANSS, MADRS) on the QLS.

**Results:**

The overall model explained 47% of variance in QLS and 17% of the variance in SOFI, but only 15% in SWN-K. Factor analysis suggested four factors: "Functioning," "Daily Living," "Depression," and "Psychopathology." A strong positive correlation was observed between the SOFI and QLS (r = 0.669), and both the QLS and SOFI loaded on the "Functioning" factor, suggesting redundancy between these scales. The measurement profiles from the categorical latent variable analysis showed significant variation in functioning and quality of life despite similar levels of psychopathology.

**Conclusions:**

Researchers should consider collecting PANSS, SOFI, and SWN-K in their trials. This would allow a broad spectrum of assessments that would have the ability to capture a wide range of treatment outcomes and allow for a rich characterization of the subgroups involved. Additional research is needed to identify the critical cognitive measures.

**Trials registration:**

Clinical trials registration: Predicting Response to Risperidone Treatment Through Identification of Early-onset of Antipsychotic Drug Action in Schizophrenia

ClinicalTrials.gov identifier: NCT00337662; http://www.clinicaltrials.gov/

## Background

Schizophrenia is a complex, multidimensional disorder. Patients diagnosed with schizophrenia exhibit positive, negative, and mood symptoms as well as experience cognitive and functional impairments. To fully assess the various dimensions affected by schizophrenia, clinical trials often include multiple scales measuring various symptom profiles, cognition, quality of life, subjective well-being, and functional impairment.

While the different measurement scales generally assess diverse aspects of schizophrenia, these scales may have some overlap in the constructs they measure. For example, the Schizophrenia Objective Functioning Instrument was designed to assess functioning, and the Quality of Life Scale was designed to assess a patient's quality of life; however, both have domains that assess occupational and psychosocial functioning. Therefore, it seems reasonable to propose that both scales would show some similarity (i.e., conceptual overlap of functioning). The use of multiple scales that result in the collection of redundant information may lead to increased study burden (e.g., multiple scales, multiple items per scale, and a 30- to 45-minute time requirement to administer each scale). Incorporating multiple assessment scales may require more time and effort from patients, additional staff time at study sites to administer measures and record data which could cause potential data quality issues, and an overall increased cost of conducting clinical trials.

Recently, we published data from a prospective clinical study in patients diagnosed with schizophrenia or a related disorder showing that patients who exhibited early response to antipsychotic treatment experienced early and consistent improvement across multiple symptom domains, subjective well-being, and health outcomes [1,2]. In this exploratory study, we used baseline data from this clinical trial to: 1) characterize the relationship among different symptomatic and functional measures; and 2) identify a more parsimonious set of measures that minimize conceptual overlap.

## Methods

### Study subjects

Data was obtained from a previously published clinical trial undertaken to assess the efficacy of early onset of antipsychotic drug action in schizophrenia [1,2]. Patients (N = 628) who entered the study were diagnosed with schizophrenia, schizoaffective disorder, or schizophreniform disorder. Patients had to be at least moderately ill at the start of the study and experiencing an exacerbation of their illness that required an intensification of the level of psychiatric care during the 2 weeks before entering the study.

The mean age of patients was 41.7 (standard deviation [SD] = 10.9) years old, the mean age of their first psychotic episode was 25.5 (SD = 9.8) years, 62% were male, 44% of the patients were Caucasian, and 45% were African American.

The original study protocol was approved by the ethical review boards responsible for individual study sites, and all patients or their legal guardians gave written, informed consent before entering the study. The study was conducted in accordance with the Declaration of Helsinki.

### Assessment scales

Patients were evaluated at baseline using several different assessment scales; key features of each measure are summarized in Table 1.

**Table 1:** Properties of Each Measure Studied

Scales	Description	No. of Items	Minutes Needed	Minutes Needed	Meaning of High Score
			**Clinician**	**Patients**	**good/bad**

PANSS	Psychopathologic symptoms	30	30-40		bad
	Negative symptoms	7			bad
	Positive symptoms	7			bad
	Disorganized thoughts	7			bad
	Hostility/excitement	4			bad
	Anxiety/depression	4			bad
MADRS	Depression	10	10		bad
BACS-SCT	Digital symbol coding test	1	3	1.5	good
SWN-K	Subjective well-being*	20		5-10	good
	Positively worded statements	10			good
	Negatively worded statements	10			bad
SOFI	Objective functioning	49	30-45		good
	Living situation	5			good
	Instrumental activity of daily living	14			good
	Productive activity	25			good
	Social functioning	5			good
QLS	Quality of life	21	30-45		good
	Common objects and activities	2			good
	Instrumental role	4			good
	Interpersonal relations	8			good
	Intr*a*psychic foundation	7			good

Abbreviations: BACS-SCT = Brief Assessment of Cognition in Schizophrenia Symbol Coding Test; MADRS = Montgomery-Asberg Depression Rating Scale; No. = number; PANSS = Positive and Negative Syndrome Scale; QLS = Quality of Life; SOFI = Schizophrenia Objective Functioning Instrument; SWN-K = Subjective Well-being Under Neuroleptics Short Form.

*For the SWN-K total score, each negatively-worded statement is reversed.

The Positive and Negative Syndrome Scale (PANSS) is a 30-item, clinician-rated instrument of positive, negative, and general psychopathology symptoms (each item scored from 1 = absent to 7 = severe; total score ranging from 30 to 210) [3]. The Montgomery-Asberg Depression Rating Scale (MADRS) is a 10-item, clinician-rated scale for severity of depressive mood symptoms (each item scored from 0 = absent to 6 = severe; total score ranging from 0 to 60) [4].

The Brief Assessment of Cognition in Schizophrenia Symbol Coding Test (BACS-SCT) is a tool for measuring attention and the speed of information processing [5].

The Subjective Well-being Under Neuroleptics Scale Short Form (SWN-K) is a 20-item, patient-rated instrument designed to capture a patient's subjective well-being (each item is scored 1 to 6; total score ranging from 20 to 120 points) [6]. This scale was originally developed with five conceptual domains using two positively worded and two negatively worded items per domain. However, in a previous study analyzing the structure of SWN-K using the same database, a 2-factor solution was obtained based on how the items were worded, namely a positively worded factor and a negatively worded factor [7].

The Schizophrenia Objective Functioning Instrument (SOFI) is a 49-item, clinician-rated instrument used to assess living situation, instrumental activities of daily living, productive activities, and social function as reported by patient, caregiver, and treatment team with each domain and overall rating scored from 1 to 100 (high scores = normal or unimpaired functioning, and low scores = severe impairment) [8].

The Quality of Life Scale (QLS) is a 21-item, semi-structured, interviewer-administered instrument that covers the dimensions of intrapsychic foundations, interpersonal relations, instrumental roles, and common objects and activities (each item is rated 0 = severe impairment to 6 = high functioning; total score ranging from 0 to 126) [9].

### Statistical analysis

#### Path analysis for relationship among measures

To explore the relationships among multiple schizophrenia measures, Pearson correlation coefficients were calculated, and structural equation modeling was used to build path models using maximum likelihood estimation. The total score of each measure was used. The PANSS and MADRS assess symptoms, and the BACS-SCT measures processing speed deficits which are assumed to be more direct or proximal manifestations of schizophrenia; therefore, the scores for these measures were designated as "exogenous variables" (predictors) in this study. The SWN-K assesses subjective well-being, and the SOFI and QLS measure functioning and quality of life; these variables are assumed to change more distally as a consequence of the proximal symptoms. Therefore, the scores for these measures were designated as "endogenous variables" (outcomes). The path parameters were fixed at 0 (i.e., arrows were deleted) for nonsignificant effects (i.e., p-value of estimated effect > 0.1), and the path parameters were freed (i.e., arrows were added) one at a time, as indicated by the largest modification index calculated by the program. The overall fit of the final model was assessed by the root mean square error of approximation (RMSEA), and the final optimal model was chosen for which the Bayesian Information Criteria (BIC) was the least.

Since the measurements were on different scales, all variables in the path model were standardized. Given that the SWN-K had been selected as having the ultimate outcome with the best model fit, a sensitivity analysis was implemented using QLS as the ultimate outcome variable with the SWN-K and SOFI as the effect mediators. Furthermore, in the path diagrams the relationships were assumed to be linear. The linear relationship between variables was checked in separate simple regression analyses by including the quadratic term in the model.

#### Exploratory factor analysis for measurement structure

To determine the overall factor structure of all the schizophrenia measures, a total of 17 variables were used, including the subdomains of the QLS, SOFI, PANSS, SWN-K, and the overall ratings for BACS-SCT and MADRS. Because we aimed to check the possible overlap and uniqueness of the measurements, an orthogonal rotation (i.e., varimax) was selected in this analysis. A variable was judged to load significantly on a single factor if the loading was at least 0.3 and at least twice its loading on any other factor. A variable was judged to cross-load when its factor loadings were greater than 0.3 on more than one factor and the greatest factor loading was not more than twice the other loadings.

#### Categorical latent variable analysis (CLVA) for measurement profile

To determine the measurement profiles, a CLVA was conducted using the same 17 variables used in the exploratory factor analysis. CLVA assumes population heterogeneity based on the analyzed measures and provides estimates for an individual's probability of membership associated with each latent class. An individual was assigned to the latent class for which the membership probability was the highest.

Diagnostic statistics, such as the log-likelihood, Akaike Information Criteria (AIC), BIC, and sample size-adjusted BIC (aBIC), were considered in deciding the number of classes. All analyses were carried out using the Mplus software program (Version 5, Muthén & Muthén, Los Angeles, CA, USA) [10].

## Results

Pearson correlation coefficients among the various measurement scales are shown in Table 2. The greatest correlation was observed between the QLS and SOFI (r = 0.669). The second greatest correlation was observed between the PANSS and SOFI (r = -0.407), followed closely by the correlation coefficients for PANSS and QLS (r = -0.387), and the MADRS and SWN-K (r = -0.354).

**Table 2:** Pearson Correlation Coefficients among Measurements

	QLS	SWN-K	SOFI	MADRS	PANSS
QLS	1				
SWN-K	0.202	1			
SOFI	0.669	0.135	1		
MADRS	-0.161	-0.354	-0.097	1	
PANSS	-0.387	-0.160	-0.407	0.281	1
BACS-SCT	0.048	0.004	0.092	0.055	-0.041

Abbreviations: BACS-SCT = Brief Assessment of Cognition in Schizophrenia Symbol Coding Test; MADRS = Montgomery-Asberg Depression Rating Scale; PANSS = Positive and Negative Syndrome Scale; QLS = Quality of Life; SOFI = Schizophrenia Objective Functioning Instrument; SWN-K = Subjective Well-being Under Neuroleptics Short Form.

Pearson correlation coefficients are based on the following scores: QLS total score, SWN-K total score, SOFI global rating, MADRS total score, PANSS total score, and BACS symbol coding test (BACS-SCT).

### Path model

**Figure 1:**
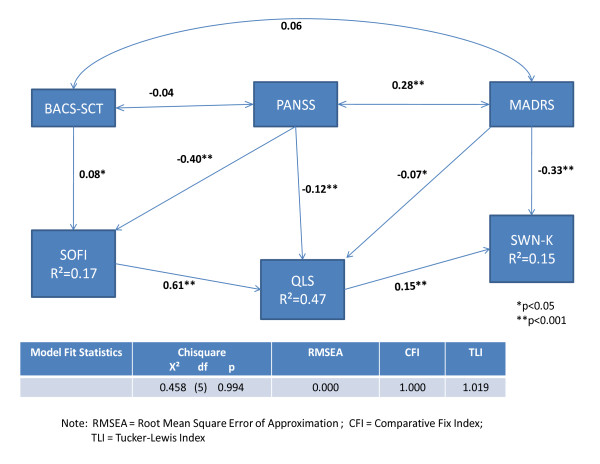
**Path Model: Standardized Effects of Predictors on Outcome**.

Figure 1 summarizes the path analysis results. The model was satisfactory in terms of the overall fit with an χ^2 ^p-value of 0.99 and a RMSEA of 0. In the optimal path model depicting relationships among the measures, the SWN-K was selected as the final outcome, while the SOFI mediated the effect of the exogenous variables on the QLS (Figure 1, Table 3). The path model explained 47% of the variance of QLS, 17% of the variance of SOFI, and 15% of the variance of SWN-K. The PANSS and MADRS were significantly correlated, while neither the PANSS nor MADRS were significantly related with BACS-SCT.

**Table 3:** Standardized Effects of the PANSS, MADRS and BACS-SCT

Exogenous Variable	Endogenous Variable		Standardized Effects	
		
		Direct	Indirect	Total
PANSS				
	SOFI	-0.40**		-0.40**
	QLS	-0.12**	-0.25**	-0.37**
	SWN-K		-0.05*	-0.05*
MADRS				
	QLS	-0.07*		-0.07*
	SWN-K	-0.33**	-0.01	-0.34**
BACS-SCT				
	SOFI	0.08*		0.08*
	QLS		0.05*	0.05*
	SWN-K		0.01	0.01

Abbreviations: BACS-SCT = Brief Assessment of Cognition in Schizophrenia Symbol Coding Test; MADRS = Montgomery-Asberg Depression Rating Scale; PANSS = Positive and Negative Syndrome Scale; QLS = Quality of Life; SOFI = Schizophrenia Objective Functioning Instrument; SWN-K = Subjective Well-being Under Neuroleptics Short Form.

*p < 0.05, **p < 0.001

The standardized effects of the exogenous variables PANSS, MADRS, and BACS-SCT on the endogenous variables SOFI, QLS and SWN-K are shown in Table 3.

The MADRS appears to be predictive of SWN-K in a direct way. One SD change of MADRS led to an expected direct change of -0.33 SD in the SWN-K. The indirect effect of MADRS on SWN-K via QLS was not significant (p > 0.05). PANSS was predictive of QLS and SOFI with a similar total effect (-0.37 SD and -0.40 SD, respectively). BACS-SCT had weak effects on the SOFI and QLS. The QLS and SOFI were highly correlated with one SD change of SOFI, which led to an expected direct change of 0.61 SD in QLS. About two-thirds of the total PANSS effect on QLS was mediated by SOFI. A sensitivity analysis (assuming QLS as the ultimate outcome and SOFI and SWN-K as the mediators) did not significantly change the variances explained by the model.

### Dimensionality of scales

The scree plot from the factor analysis suggested that the eigenvalues are greater than one until the fifth eigenvalue: eigenvalue = 5.15, 1.93, 1.57, 1.13, and 0.95 for factors 1 through 5, respectively. In addition, all the loadings were below 0.3 on one factor when a 5-factor solution was selected, so the 4-factor solution was adopted.

Table 4 shows the results of the factor analysis across the 17 variables. Four factors were identified. The "Functioning" factor included all four subdomains of the QLS, the instrumental activity of daily living, productive activity, and social functioning of the SOFI, and the negative symptoms factor of the PANSS. The "Daily Living" factor included all 4 subdomains of the SOFI, with the SOFI instrumental activity of daily living subdomain loading most highly. Several SOFI subscales cross loaded on both the "Functioning" and "Daily Living" factors. The "Depression" factor included the MADRS, the negatively worded statements of the SWN-K, and the depression/anxiety factor of the PANSS. The "Psychopathology" factor included 4 of the 5 PANSS factors-negative symptoms; positive symptoms; disorganized thoughts; and hostility/excitement. The BACS-SCT and the positively worded statements of the SWN-K did not load on any identified factor.

**Table 4:** Factor Analysis of Scales

	Functioning	Daily Living	Depression	Psychopathology
QLS				
Common objects and activities	**0.61**	0.29	0.04	0.22
Intr*a*psychic foundation	**0.80**	0.24	0.05	0.20
Interpersonal relations	**0.70**	0.16	0.15	0.05
Instrumental role	**0.59**	0.17	0.04	0.00
MADRS	-0.11	0.04	**-0.69**	-0.08
SOFI				
Living situation	0.24	**0.54**	-0.02	0.20
Instrumental activity of daily living	**0.40**	**0.78**	0.01	0.24
Productive activity	**0.48**	**0.56**	0.05	0.14
Social functioning	**0.57**	**0.54**	0.12	0.14
SWN-K				
Positively worded statements	0.23	0.02	0.22	0.00
Negatively worded statements	-0.08	-0.04	**-0.43**	0.02
PANSS				
Negative symptoms	**-0.37**	-0.12	-0.16	**-0.40**
Positive symptoms	-0.13	-0.05	-0.13	**-0.44**
Disorganized thoughts	-0.24	-0.10	0.09	**-0.94**
Hostility/excitement	0.01	-0.10	-0.17	**-0.35**
Anxiety/depression	0.02	0.01	**-0.80**	-0.16
BACS-SCT	-0.02	0.11	-0.06	0.20

Abbreviations: BACS-SCT = Brief Assessment of Cognition in Schizophrenia Symbol Coding Test; MADRS = Montgomery-Asberg Depression Rating Scale; PANSS = Positive and Negative Syndrome Scale; QLS = Quality of Life; SOFI = Schizophrenia Objective Functioning Instrument; SWN-K = Subjective Well-being Under Neuroleptics Short Form.

Bolded text indicates significant loading or cross-loading.

### Measurement profiles

Table 5 summarizes the model-fit statistics for the categorical latent variable analyses with 1 to 4 latent classes. A 3-class model stood out as the optimal solution in terms of the highest likelihood and the smallest AIC, BIC, and aBIC. In addition, a 3-class solution appeared to provide a fair interpretation of the population with a reasonable percentage in each class.

**Table 5:** Model-Fitting Statistics for Measurement Profile (Categorical Latent Variable Analyses)

Number of Class	1	2	3	4
Log likelihood	-37759	-36834	-36004	-36425
BIC	75629	73838	72459	73137
aBIC	75736	74003	72237	73417
AIC	75586	73772	72148	73026

Abbreviations: AIC = Akaike Information Criteria; aBIC = Adjusted Bayesian Information Criteria; BIC = Bayesian Information Criteria.

**Figure 2:**
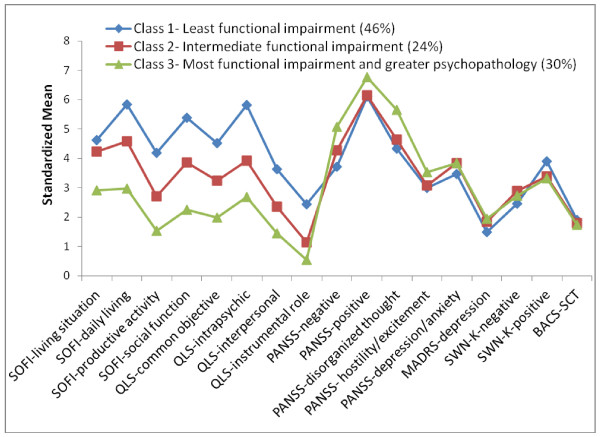
**Measurement Profiles**. Abbreviations: BACS-SCT = Brief Assessment of Cognition in Schizophrenia Symbol Coding test; MADRS = Montgomery-Asberg Depression Rating Scale; PANSS = Positive and Negative Syndrome Scale; QLS = Quality of Life; SOFI = Schizophrenia Objective Functioning Instrument; SWN-K = Subjective Well-being Under Neuroleptics Short Form.

Figure 2 summarizes the 3-class measurement profiles. Since the scale ranges vary greatly, the standardized means were used in the graph. Patients in Class 1 (46%) showed the least functional impairment on the QLS and SOFI, and moderate severity in psychopathology on the PANSS. Patients in Class 2 (24%) showed moderate functional impairment on the QLS and SOFI, and moderate severity in psychopathology on the PANSS. Patients in Class 3 (30%) showed the greatest functional impairment on the QLS and SOFI, and the greatest severity in psychopathology on the PANSS.

## Discussion

In this exploratory study, we aimed to quantitatively characterize the relationships among clinical, functional, cognitive, and quality-of-life measures, and to identify a more parsimonious set of measurements. In the optimal path model, the SWN-K was selected as the ultimate outcome, although the overall model explained only 15% of variance in the SWN-K. The strongest correlation was observed between the SOFI and the QLS. In the path model, the effect of the PANSS (an exogenous variable) on the QLS was mediated primarily by the SOFI. The factor analysis suggested four factors: "Functioning" (loading by SOFI, QLS, and PANSS negative), "Daily Living" (loading by SOFI), "Depression" (loading by MADRS, PANSS anxiety/depression factor, and SWN-K negatively-asked questions), and "Psychopathology" (loading by PANSS negative and positive symptoms, disorganized thoughts, and hostility/excitement factors). In addition, the measurement profile analysis revealed three classes that generally followed a pattern from less severely impaired on measures of functioning (QLS, SOFI) and less severely ill (PANSS) (Class 1) to moderately impaired on functioning (Class 2), and most severely impaired on functioning and psychopathology (Class 3).

Our findings suggest that redundancy exists among the measures studied, particularly among the clinician-rated functional and quality of life measures. The Pearson correlation coefficients among measurements revealed the strongest correlation between the SOFI and the QLS (r = .669). Previously, during development and validation of the SOFI, psychometric properties also revealed a moderate correlation between the SOFI and QLS, similar in magnitude to the one observed in the current study with a correlation coefficient of r = .61 for the patient-rated version of the SOFI and r = .52 for the informant version [8]. In addition, the path model explained 47% of the variance in the QLS, and a majority of this effect was mediated by SOFI, suggesting overlap between QLS and SOFI. Furthermore, while the QLS loaded on "Functioning," the SOFI loaded on both the "Functioning" and "Daily Living" factors. While both the SOFI and QLS provide, to some degree, a measure of social and occupational functioning, the SOFI provided a broader measure of outcomes that included functioning and daily living.

The best-fit model for the path analysis, which selected the SWN-K to be the ultimate outcome, revealed that only 15% of the variance in SWN-K could be explained by the model, which included measures of symptoms, functioning, and cognition. This finding may suggest that the SWN-K is a unique measure capturing potential treatment effects not captured by the other measurement scales. The SWN-K negatively worded statements loaded on the "Depression" factor, while the SWN-K positively worded statements did not meet the factor-loading criteria. This latter finding may suggest important differences between these two components of the SWN-K, a finding that was consistent with a recent factor and item response theory analysis on the English version of SWN-K [7]. Additional work will be necessary to further understand the relationship of the positively and negatively worded statements to the psychometric properties of the scale as a whole, and the unique qualities of the SWN-K to overall treatment responsiveness.

The SWN-K total score has been demonstrated previously to be associated with dopaminergic D2 receptor blockade [11], medication adherence [12], and the likelihood of achieving enduring symptomatic remission [13]. Subjective well-being has also been associated with depression. In patients with schizophrenia, depressive symptoms were significantly associated with subjective well-being in newly admitted patients [14] and during the course of acute treatment with atypical antipsychotics [15]. In these studies, a significant negative correlation was observed between the SWN-K score, the PANSS depression factor score, and the subjectively-rated Beck Depression Inventory (BDI) [14,15], although only the correlation between the SWN-K and BDI was significant following 8 weeks of treatment [15]. These findings are consistent with the factor analysis in which the SWN-K negatively worded statements loaded on the "Depression" factor. In the path analysis, the physician-rated MADRS was predictive of the SWN-K in a direct fashion, with a one SD change of the MADRS leading to a change of -0.33 SD in the SWN-K. In addition, the correlation analysis had revealed a small to moderately sized negative correlation between the MADRS and SWN-K (r = -0.35). These findings collectively highlight the important role that depressive symptoms may play in low subjective well-being, and the importance of a patient's subjective well-being to treatment outcomes, including medication adherence and remission.

The measurement profiles of the study population detected heterogeneity primarily in measurements of social and occupational functioning and daily living activities via the SOFI and QLS, whereas the study population was generally homogeneous in psychopathological symptoms, as defined by the study inclusion criteria. Patients in Class 3 stood out as having the worst functioning and daily living and the worst or most severe symptoms. Patients in Classes 1 and 2 had moderately severe symptoms, with patients in Class 1 having the best functioning and daily living, while patients in Class 2 had moderately impaired functioning and daily living. Patients in Class 1 with the best functioning and daily living also seemed to show somewhat higher scores on SWN-K positively worded statements.

The BACS-SCT, or symbol coding test, is a measure of attention and speed of information processing [5]. A recent meta-analysis of 37 studies comparing digit symbol coding tasks to other cognitive measures in schizophrenia demonstrated a significantly larger mean effect size for impairment in digit symbol coding compared with the effects of impairment in episodic memory, executive function, and working memory, suggesting that information processing inefficiency is a central feature of the cognitive deficit in schizophrenia [16]. A subsequent study examining the predictive relationships between neuropsychological domains, functional competence, social competence, symptoms, and real-world behavior demonstrated that only processing speed had both direct and indirect effects on all three real-world behaviors including domains of work skills, interpersonal relationships, and community activities [17]. Reduced processing speed has been associated with functional disability observed in patients with schizophrenia [18,19]. In previous research, we found information processing speed had both direct and indirect effects via negative symptoms on three domains of functioning, as measured by the QLS at baseline and following 24 weeks of antipsychotic treatment [20].

In the current analysis, the BACS-SCT did not play a major role in any of the current analyses including the path-modeling and factor analysis. Previously, we used a composite measure of processing speed that included an average of two subscales including digit symbol coding and the verbal fluency scale, and that focused on QLS domains of functioning as the ultimate outcome [20]. Perhaps the use of only the digit symbol coding test underlies the different findings. It would be legitimate to argue that the limited role of the BACS-SCT observed in these analyses suggests that this test may also be capturing unique information. However, in contrast to the SWN-K, which showed a mild to moderate correlation with the QLS, SOFI, and MADRS, the BACS-SCT was not significantly related to any of the clinical or functional measures evaluated in this study.

Our findings from the path analysis using structural equation modeling, the factor analysis of the measurement structure, and the measurement profiles from the latent class analysis complement each other in understanding the measurements. Though each model was implemented under different assumptions, the findings that QLS and SOFI measures were highly correlated was consistent. The MADRS and SWN-K were also correlated, while the BACS-SCT was not significantly related with any of the other measures. This study may contribute to the effort to better understand schizophrenia measurements with the goal of identifying a parsimonious data set.

There were several limitations to the current analyses. First, patients had to have a particular level of acuity to enter the study, and this likely restricted the possible range of baseline scores on the PANSS. Second, for the path analysis, an assumption was made that the PANSS, MADRS, and BACS-SCT were "exogenous variables" assessing symptoms and attention and processing speed deficits assumed to be more proximal to disease manifestation. Additionally, it was assumed that the SWN-K, SOFI, and QLS were "endogenous variables" assessing subjective well-being, functioning, and quality of life thought to be the consequences of the proximal symptoms. However, our previous work has demonstrated that subjective well-being, functioning, and quality of life can change as early as 2 weeks into treatment and seemingly mirror improvements in symptoms [2]. Thus, the temporal relationship of change among these variables is not fully understood, and the outcomes observed are thereby limited by the proposed relationships set forth by the specifications of the statistical models. Third, we incorporated the SWN-K total score in the correlation and path analyses and the SWN-K positively worded and negatively worded statements in the factor analysis and measurement profiles. Therefore, comparisons for the SWN-K total score cannot be made across all of the analyses.

This study was exploratory in nature, with the results being driven by both statistics and knowledge of the disease and population. Further, even a perfect fit of the model would not prove that the inferences are causal, but merely suggest that the model fits the data well. It would be helpful to attempt to replicate the results for a similar population at a different time, and/or to replicate the results in a different patient population with similar or varying disease characteristics. It is important to realize that results of this study reflect a chronically ill patient population moderately to severely ill with an exacerbation of symptoms, and the observations made are dependent upon the scales incorporated in the study design and assessed at baseline.

## Conclusions

Researchers should consider collecting PANSS, SOFI, and SWN-K in their trials. This would allow a broad spectrum of assessments that would have the ability to capture a wide range of treatment outcomes and allow for a rich characterization of the subgroups involved. Additional research is needed to identify the critical cognitive measures.

## Competing interests

LC, JJ, BJK, HAS are employees and current shareholders of Eli Lilly and Company. SKW is an employee and current shareholder of Lilly USA, LLC, a subsidiary of Eli Lilly and Company. GP is former employee and current shareholder of Eli Lilly and Company, and a current employee of Sunovion Pharmaceuticals. PS is an employee of the University of Cincinnati, College of Medicine. DN is member of advisory boards of Eli Lilly, Janssen Cilag, Lundbeck and Servier. He has received honoraria from Astra Zeneca, Otsuka and Roche.

## Authors' contributions

LC and GP conceived the study and contributed to the initial design and coordination. LC performed the statistical analysis, and wrote the initial draft of the manuscript. SKW coordinated the development of subsequent drafts, including incorporation of revisions to each new version. All authors participated in the analysis and interpretation of the data, and revising the manuscript for critically important intellectual content. In addition, all authors read and approved the final version of the manuscript.

## Pre-publication history

The pre-publication history for this paper can be accessed here:

http://www.biomedcentral.com/1471-244X/11/203/prepub
